# Diverse *Bradyrhizobium* spp. with Similar Symbiosis Genes Nodulate Peanut in Different Regions of China: Characterization of Symbiovar sv. Arachis

**DOI:** 10.3390/plants12213776

**Published:** 2023-11-06

**Authors:** Junjie Zhang, Yufeng Feng, Jingqi Wang, Entao Wang, Mitchell Andrews

**Affiliations:** 1 College of Food and Bioengineering, Zhengzhou University of Light Industry, Zhengzhou 450002, China; 13598051592@163.com (Y.F.); wjq262023@163.com (J.W.); 2 Collaborative Innovation Center for Food Production and Safety of Henan Province, Zhengzhou 450002, China; 3 Departamento de Microbiología, Escuela Nacional de Ciencias Biológicas, Instituto Politécnico Nacional, Ciudad de México 11340, Mexico; entaowang@yahoo.com.mx; 4 Faculty of Agriculture and Life Sciences, Lincoln University, Lincoln 7647, New Zealand

**Keywords:** *Arachis hypogaea*, *Bradyrhizobium* symbiovar, MLSA, *nod* genes, *nodC*

## Abstract

A total of 219 rhizobial strains isolated from peanut grown in soils from six peanut croplands in Zhengyang county, Henan Province, were typed by PCR-RFLP of IGS sequences. Their phylogenetic relationships were refined on representative strains using sequence analyses of 16S rRNA genes, housekeeping genes (*atpD*, *recA*, *glnII*) and symbiosis genes (*nodA*, *nodC* and *nifH*). The 219 rhizobial isolates were classified into 13 IGS types, and twenty representatives were defined within eight *Bradyrhizobium* genospecies: *B. guangdongense* covering 5 IGS types (75.2% of total isolates), *B. guangzhouense* (2 IGS types, 2.7% total isolates), *B. zhengyangense* (1 IGS type, 11.3% total isolates) and five novel genospecies (5 IGS types, 0.9 to 3.2% total isolates). All representative strains had identical *nodA*, *nodC* and *nifH* sequences except for one *nifH* sequence. With this one exception, these sequences were identical to those of the type strains of *Bradyrhizobium* species and several *Bradyrhizobium* genospecies isolated from peanut in different regions of China. The *nodC* sequences of all strains showed < 67% similarity to the closest strains on the Genbank database indicating that they are representative of a novel *Bradyrhiobium* symbiovar. This study has shown that (1) diverse *Bradyrhizobium* spp. with similar symbiosis genes nodulate peanut in different regions of China. (2) Horizontal transfer of genes involved in nodulating peanut is common between *Bradyrhizobium* species in soils used to grow the crop in China. (3) The strains studied here are representative of a novel *Bradyrhizobium* symbiovar that nodulates peanut in China. We propose the name sv. arachis for this novel symbiovar indicating that the strains were isolated from *Arachis hypogaea*. Results here have practical implications in relation to the selection of rhizobial inoculants for peanut in China.

## 1. Introduction

Legume species show specificity for rhizobial symbiont [1]. Mechanisms that maintain this specificity operate at different points in the nodulation process including during communication between the two organisms in the soil [2]. Generally, the nodulation process is initiated by the legume production of a mix of compounds, mainly flavonoids, into the rhizosphere. Different legume species produce different types and mixes of compounds that are specific to particular rhizobia. These compounds are absorbed by compatible rhizobia in the soil, and this activates their nodulation protein D (NodD) by stimulating the binding of NodD to *nod* gene promoters. The NodD protein triggers the transcription of a range of genes within the rhizobium including those required to produce Nod factors, the signal molecules from the rhizobium that induce nodule morphogenesis in the legume. These genes include *nodABC* that encode the enzymes required for the synthesis of the core Nod factor structure of an N-acetyl glucosamine oligosaccharide backbone with a fatty acyl chain at the non-reducing end. Nod factors differ in their length of the N-acetyl glucosamine oligosaccharide backbone and length and saturation of the fatty acid chain. Also, *nod* genes in addition to *nodABC* can encode species-specific substitutions to the Nod factor core. Specific *nod* genes, but *nodC* in particular, have been shown to be closely correlated with legume host specificity [3,4].

The symbiosis (*sym*) genes that enable rhizobial strains to induce N_2_-fixing nodules on legumes (*nod*, *nif* and *fix* genes) can be gained or lost as they are encoded on transferable plasmids or symbiosis islands. Horizontal transfer of *sym* genes can occur between, and more commonly within, bacterial genera [5,6]. Thus, strains of different rhizobial species within and across genera can have similar *sym* genes. Also, different strains within a particular rhizobial species can have different *sym* genes. The term symbiovar (sv.) describes strains of the same or different rhizobial species that are able to establish symbiosis with distinct legume species and can be distinguished by specific symbiotic gene phylogenies [3,4,7].

Cultivated peanut (*Arachis hypogaea* L.) originated and was domesticated in South America, but it is now an important grain and oil legume crop in many countries, with China the largest producer worldwide [8]. Often, crop legumes grown outside their normal range require inoculation with compatible rhizobia, but in China, peanut crops are not inoculated and rely on soil populations for nodulation. Previous studies in China reported peanut to be nodulated exclusively by *Bradyrhizobium* species and mainly native strains. *Bradyrhizobium lablabi* [9], *B. arachidis* [10], *B. guangdongense*, *B. guangxiense*, *B. guangzhouense*, *B. nanningense*, *B. zhanjiangense* [11,12] and *B. zhengyangense* [13] were isolated from peanut in different regions of China and formally described. Henan Province in central China has become the largest area for peanut production in China, with over 1.47 million hectares. Zhang et al. [14] characterized 217 strains isolated from peanut grown in soils from six field sites in the Nanyang city, administrative area, Henan Province. Phylogenetic analysis of *recA*, *atpD* and *glnII* genes of representative strains identified *B. guangdongense*, *B. ottowaense* and three novel *Bradyrhizobium* genospecies. Attempts to sequence the *nodC* gene of the isolates were unsuccessful. However, their *nodA* and *nifH* gene sequences were not congruent with the core genes on phylogenetic analysis and most strains showed sequences similar to those of *Bradyrhizobium* spp. isolated from peanut in southeast China. This indicates that horizontal transfer of *nodA* and *nifH* genes occurred between *Bradyrhizobium* in soils of peanut crops.

In the current study, the diversity of rhizobia-nodulating peanut in Henan Province was explored further. A total of 219 rhizobial strains isolated from peanut grown in soils from six peanut croplands in Zhengyang county were typed by PCR-RFLP of IGS sequences. Their phylogenetic relationships were refined on representative strains using sequence analyses of 16S rRNA genes, housekeeping genes (*atpD*, *recA*, *glnII*) and symbiosis genes (*nodA*, *nodC* and *nifH*). A new symbiovar of peanut is described.

## 2. Results

### 2.1. PCR-Based RFLP Analysis of IGS

A total of 219 peanut rhizobial strains were isolated in this study that separated into 13 IGS types (Table 1). IGS type 2 was the most prevalent with a total of 155 strains representing 69.82% of all isolates and occurred in all sites. IGS type 1 also occurred in all sites, with a total of 25 strains representing 11.26% of all isolates (Table 1). The remaining 11 IGS types occurred at one (IGS Types 6, 7, 9–13) or two (IGS types 3–5 and 8) sites. The number of different IGS types detected at each site varied from 2 (site ZD) to 8 (site ZA). 

### 2.2. Phylogenetic Analysis of 16S rRNA and Housekeeping Genes

**Figure 1:**
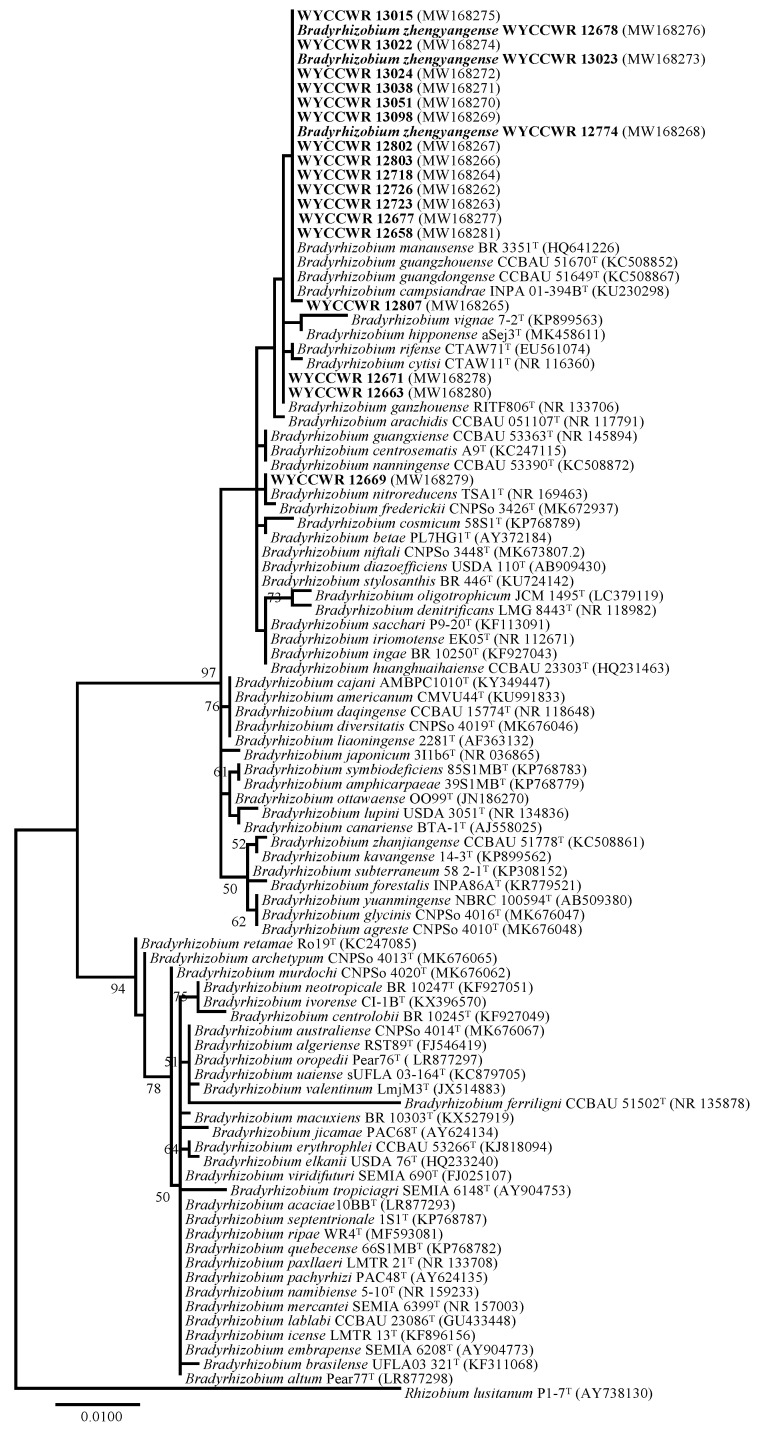
Maximum-likelihood phylogenetic tree based on 16S rRNA gene sequences showing the relationships of rhizobia isolated from *Arachis hypogaea* L. grown in soil from Zhengyang, Henan Province. The tree was constructed under a T92+G model. Bootstrap confidence values > 50% are indicated at the internodes. Bar = 1% nucleotide divergence. GenBank accession numbers are shown in brackets. The bold strains are the representatives of the isolates in this study.

In 16S rRNA gene phylogeny, the 20 representative isolates formed a single and well-supported clade (97% bootstrap value) together with the type strains of 44 defined *Bradyrhizobium* species (Figure 1). The isolates separated into three subclades within this clade. Seventeen isolates showed identical or highly similar 16S rRNA sequences to *B. guangdongense* CCBAU 51649^T^ and *B. guangzhouense* CCBAU 51670^T^ isolated from peanut in Guangdong province, Southeast China [11,12], and *B. manausense* BR 3351^T^ and *B campsiandrae* INPA 01-394B^T^ (=UFLA 01-1174^T^) isolated from *Vigna unguiculata* and *Campsiandra laurilifolia*, respectively, in the Amazon region, Brazil [15,16]. Two isolates (WYCCWR 12671 and WYCCWR 12663) showed identical 16S rRNA sequences to *B. ganzhouense* RITF806^T^ isolated from *Acacia melanoxylon* in Jiangxi Province, Southeast China [17]. The final strain (WYCCWR 12669) was most closely related to *B. nitroreducens* TSA1^T^ isolated from rice paddy soil in Japan [18].

**Figure 2:**
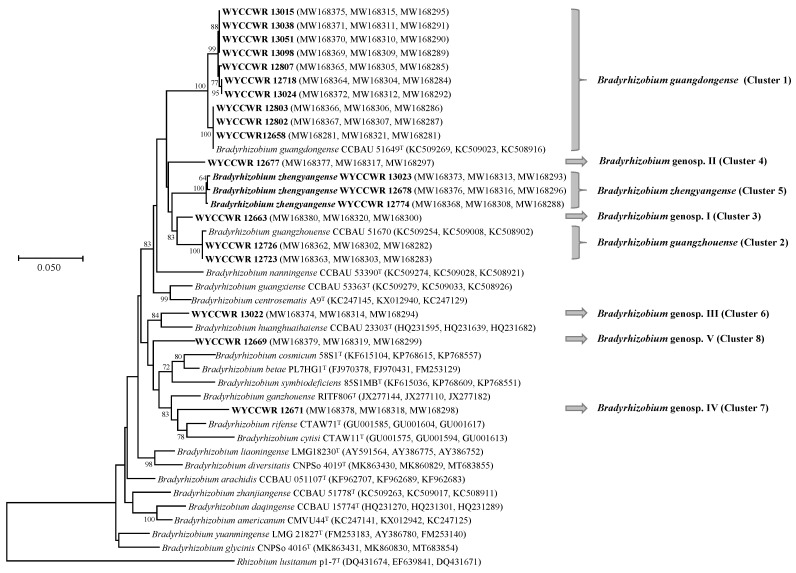
Maximum-likelihood phylogenetic tree based on concatenated *recA-glnII-atpD* gene sequences showing the relationships of rhizobia isolated from *Arachis hypogaea* L. grown in soil from Zhengyang, Henan Province. The tree was constructed under a GTR+G+I model. Bootstrap confidence values >50% are indicated at the internodes. Bar = 5% nucleotide divergence. The bold strains are the representatives of the isolates in this study.

In the phylogeny of concatenated sequences of *recA* (350 bp), *glnII* (441 bp) and *atpD* (394 bp), the 20 representative strains formed eight clusters (genospecies) at a 97.0% similarity cut-off (Figure 2, Table 1). Similar grouping results were observed in the phylogenies for individual genes (Figures S1–S3). Cluster 1 (C1) contained ten representative strains of IGS types 2, 5, 9, 10 and 12, covering 167 isolates (75.2% of total isolates) that shared 98.5–100% concatenated sequence similarities with *B. guangdongense* CCBAU 51649^T^. Cluster 2 (C2) included the representative strains of IGS types 3 and 11 consisting of 6 isolates (2.7% of total), and their concatenated sequences showed 99.7% similarities with that of *B. guangzhouense* CCBAU 51670^T^. The three strains in Cluster 5, WYCCWR 13023^T^, WYCCWR 12678 and WYCCWR 12774 (IGS type 1) were formally described as a novel species *B. zhengyangense* in a separate study (13). Clusters 3 (IGS 13, 2 strains), 4 (IGS 4, 5 strains), 6 (IGS 8, 7 strains), 7 (IGS 7, 3 strains) and 8 (IGS 6, 4 strains) showed less than 96.7% similarities in MLSA with the defined *Bradyrhizobium* species. Based on these results, C1, C2 and C5 were affiliated to *B. guangdongense*, *B. guangzhouense* and *B. zhengyangense*, respectively, while clusters 3, 4, 6, 7 and 8 were considered as separate *Bradyrhizobium* genospecies. 

### 2.3. Phylogenies of Symbiosis Genes

**Figure 3:**
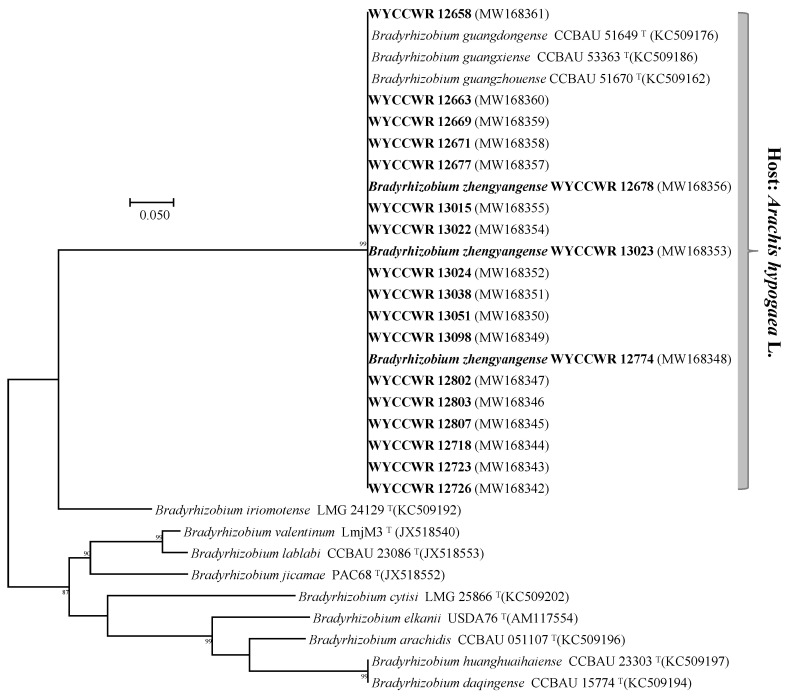
Maximum-likelihood phylogenetic tree based on *nodA* gene sequences showing the relationships of rhizobia isolated from *Arachis hypogaea* L. grown in soil from Zhengyang, Henan Province. The tree was constructed using the T92+I model. Bootstrap confidence values > 50% are indicated at the internodes. Bar = 5% nucleotide divergence. The bold strains are the representatives of the isolates in this study.

**Figure 4:**
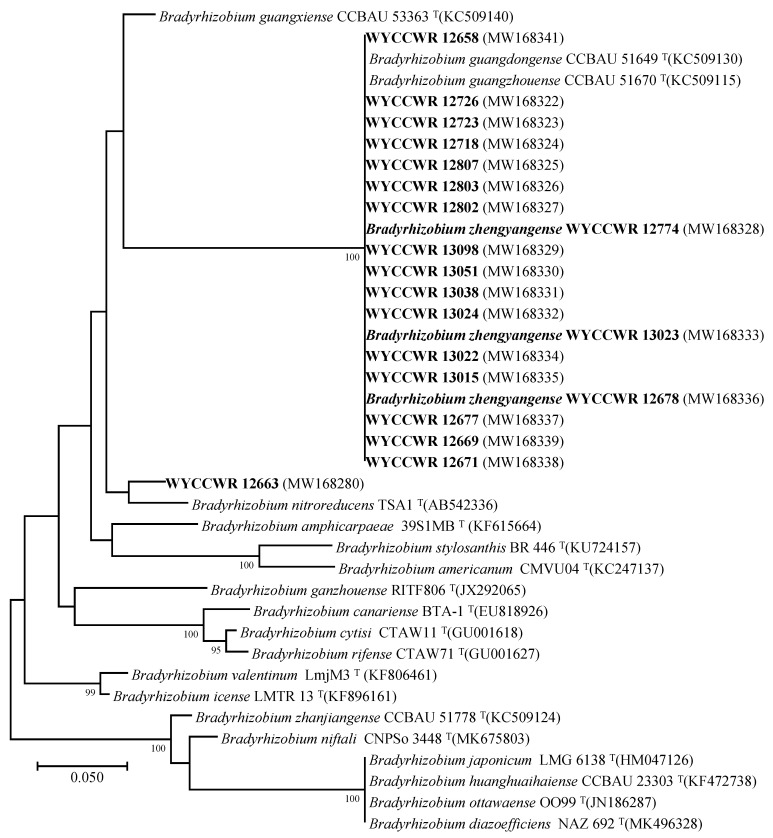
Maximum-likelihood phylogenetic tree based on *nifH* gene sequences showing the relationships of rhizobia isolated from *Arachis hypogaea* L. grown in soil from Zhengyang, Hennan Province. The tree was constructed using the T92+G model. Bootstrap confidence values >70% are indicated at the internodes. Bar = 5% nucleotide divergence. The bold strains are the representatives of the isolates in this study.

**Figure 5:**
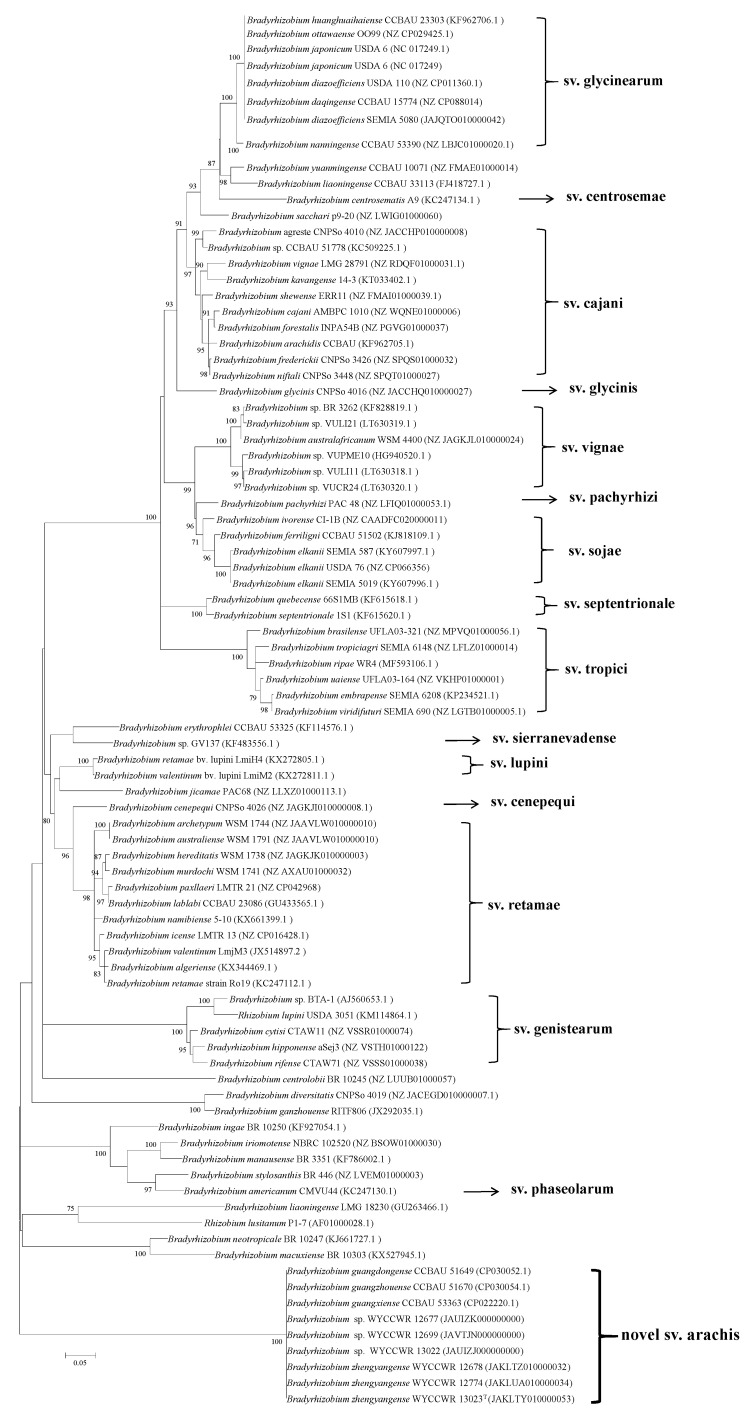
Maximum-likelihood phylogenetic tree based on *nodC* gene sequences showing the relationships of rhizobia isolated from *Arachis hypogaea* L. grown in soil from Zhengyang, Henan Province. The tree was constructed using the T92+I model. Bootstrap confidence values > 50% are indicated at the internodes. Bar = 5% nucleotide divergence. Different symbiovars of *Bradyrhizobium* were analyzed in this tree and the novel sv. arachis is proposed.

In the phylogenetic tree of the *nodA* gene, the 20 representative strains formed a unique branch together with *B. guangdongense* CCBAU 51649^T^ and *B. guangzhouense* CCBAU 51670^T^ isolated from peanut in Guangdong Province, southeast China, and *B. guangxiense* CCBAU 53363^T^ isolated from peanut in Guangxi Province, south China [11,12] which showed 100% sequence (440 bp) similarities (Figure 3). In the *nifH* phylogenetic tree, all isolates except WYCCWR 12663 were identical to *B. guangdongense* CCBAU 51649^T^ and *B. guangzhouense* CCBAU 51670^T^ (Figure 4). Strain WYCCWR 12663 aligned closest to *B. nitroreducens* TSA1^T^ (95.3% similarity) in the *nifH* phylogenetic tree. The *nodC* sequences of strains WYCCWR 13022, WYCCWR 12699 and WYCCWR 12677, three *B. zhengyangense* strains including WYCCWR 13023^T^, WYCCWR 12678 and WYCCWR 12774, *B. guangxiense* CCBAU 53363^T^, *B. guangzhouense* CCBAU 51670^T^ and *B. guangdongense* CCBAU 51649^T^ were identical and clearly separated from other *Bradyrhizobium* species (Figure 5 and Figure S4). The *nodC* sequences of all strains studied showed 66.0% and 63.2% similarity to *B. neotropicale* BR 10247^T^ and *B. macuxiense* BR 10303^T^, respectively, the two closest related *Bradyrhizobium* type strains on *nodC* phylogeny (Figure 5).

## 3. Discussion

Zhang et al. [14] reported that 217 rhizobial isolates from peanut grown in soils from six field sites in Nanyang city administrative area, Henan province, central China, separated into eight IGS types. Phylogenetic analysis of *recA*, *atpD* and *glnII* genes of representative strains of these IGS types identified *B. guangdongense*, *B. ottowaense* and three novel *Bradyrhizobium* genospecies. In the current study, a collection of 219 rhizobial isolates from peanut grown in soils from six field sites in Zhengyang county, Henan province, identified 13 IGS types. Here, phylogenetic analysis of *recA*, *atpD* and *glnII* genes of representative strains identified *B. guangdongense*, *B. guangzhouense* and six genospecies in the genus *Bradyrhizobium*. Three strains of one of these genospecies were formally described as *B. zhengyangense* [13]. It is likely that at least some of the novel genospecies reported here and by Zhang et al. [14] are representative of other novel *Bradyrhizobium* spp., but further work is required to show that this is the case [13]. Data obtained in the current study are consistent with previous reports that in China, peanut is exclusively nodulated by *Bradyrhizobium* species and mainly native strains. They also show that peanut is nodulated by diverse *Bradyrhizobium* spp. in Henan province. This is also the case in Guangdong province, southeast China, another major area of peanut production [19]. Outside China, peanut is mainly, but not exclusively, nodulated by *Bradyrhizobium* spp. [1].

*B. guangdongense* was the most common isolate identified here and in the study of Zhang et al. [14]. This species is also a common rhizobial symbiont of peanut in soils of Guangdong province [11]. This finding of the same *Bradyrhizobium* peanut-nodulating species in Henan province and the distant Guangdong province could be related to the dispersion of cultivated peanut from southeast to central China. Shao et al. [20] found that *B. liaoningense* and *B. ottawaense* were the dominant peanut-nodulating species in Shandong Province that is adjacent to Henan Province. Therefore, biogeographic differences exist in the *Bradyrhizobium* peanut-nodulating species in China.

Zhang et al. [14] reported that the *nodA* and *nifH* gene sequences of peanut rhizobia from Henan province were not congruent with the core genes on phylogenetic analysis and most strains showed sequences similar to those of *Bradyrhizobium* spp. isolated from peanut in south/ southeast China. In particular, the *nodA* gene sequences of seven of the 11 representative strains of the eight IGS types assigned to *B. guangdongense* and *Bradyrhizobium* genospecies 2 and 3 were identical to those of *B. guangdongense* CCBAU 51649^T^, *B. guangxiense* CCBAU 53363^T^ and *B. guangzhouense* CCBAU 51670^T^, all of which were isolated from peanut in south/southeast China [11,12]. The *nifH* sequences for these seven strains were identical to those of *B. guangdongense* CCBAU 51649^T^ and *B. guangzhouense* CCBAU 51670^T^. Similarly, in the current study, the *nodA* gene sequences of the 20 representative strains formed a unique branch together with *B. guangdongense* CCBAU 51649^T^, *B. guangzhouense* CCBAU 51670^T^ and *B. guangxiense* CCBAU 53363^T^ which showed 100% sequence (440 bp) similarities and in the *nifH* phylogenetic tree, all isolates except WYCCWR 12663, were identical to *B. guangdongense* CCBAU 51649^T^ and *B. guangzhouense* CCBAU 51670^T^. Also, the *nodC* sequences of strains WYCCWR 13022, WYCCWR 12699 and WYCCWR 12677, three *B. zhengyangense* strains including WYCCWR 13023^T^, WYCCWR 12678 and WYCCWR 12774, *B. guangxiense* CCBAU 53363^T^, *B. guangzhouense* CCBAU 51670^T^ and *B. guangdongense* CCBAU 51649^T^ were identical and clearly separated from other *Bradyrhizobium* species (Figure 5 and Figure S4). This indicates that horizontal transfer of *nodA*, *nodC* and *nifH* genes involved in nodulating peanut is common between *Bradyrhizobium* species in soils used to grow the crop in China [5,6].

The term symbiovar describes a group of rhizobial strains supported by similar phylogeny of symbiosis genes and host range. The current study and that of Zhang et al. [14] show that four formally described *Bradyrhizobium* species (*B guangdongense* CCBAU 51649^T^, *B. guangxiense* CCBAU 53363^T^, *B. guangzhouense* CCBAU 51670^T^ and *B. zhengyangense* WYCCWR 13023^T^) and seven *Bradyrhizobium* genospecies have similar *nodA*, *nodC* and *nifH* gene phylogenies. Host range was not tested in either study. However, *B guangdongense* CCBAU 51649^T^, *B. guangxiense* CCBAU 53363^T^, *B. guangzhouense* CCBAU 51670^T^ and *B. zhengyangense* WYCCWR 13023^T^ show a similar host range in that they produce functional nodules on peanut and *Lablab purpureus*, either do not nodulate or produce ineffective nodules on *Phaseolus vulgaris* and *Glycine max*, and do not nodulate *Medicago sativa*, *Trifolium repens* and *Vigna radiata* [11,12,13] (unpublished data). Thus, phylogeny of symbiosis genes and host range of associated type strains indicate that the strains studied here represent a new symbiovar of *Bradyrhizobium* that nodulates peanut in China. Delamuta et al. [3] proposed that a similarity of *nodC* gene sequences of approximately 92.5% or less could be used for defining new symbiovars. Here, the *nodC* gene sequences of all strains tested in the current study showed <70% similarity to the closest strains on *nodC* phylogeny indicating a novel *Bradyrhizobium* symbiovar that nodulates peanut in China. 

In summary, this study has shown that (1) diverse *Bradyrhizobium* spp. with similar symbiosis genes nodulate peanut in different regions of China. (2) Horizontal transfer of genes involved in nodulating peanut is common between *Bradyrhizobium* species in soils used to grow the crop in China. (3) The strains studied here are representative of a novel *Bradyrhizobium* symbiovar that nodulates peanut in China. We propose the name sv. arachis for this novel symbiovar indicating that the strains were isolated from peanut, *Arachis hypogaea*. The findings here have practical implications in relation to the selection of rhizobial inoculants for peanut in China. As a first step, further work will determine if the different genospecies studied here are adapted to and are more competitive in specific soils/soil conditions with the objective of producing specific peanut *Bradyrhizobium* inoculants for particular soil types. 

## 4. Materials and Methods

### 4.1. Soil Sampling and Physicochemical Characterization

Soil samples were collected from six peanut croplands where the peanut variety Yuanza 9102 is frequently cultivated, in Zhengyang County (N32°22′23″–N32°40′52″, E114°18′59″–E114°29′30″), Henan province, central China. The sampling sites covered four soil types: fluvo-aquic soil, paddy soil, Shajiang black soil and yellow cinnamon soil. In all sites, peanut had been repeatedly cropped over a period extending from June to September for more than two decades, in rotation with winter wheat (*Triticum aestivum* L.) each year, except at site ZB, where a two-year rotation consisting of peanut/wheat and green manure (*Astragalus sinicus* L.)/rice (*Oryza sativa* L.) was applied. No rhizobial inoculation was carried out in the studied fields, as is the case for the whole of Zhengyang County. Soils were collected at a depth of 10 to 20 cm from each site, during the peanut full-bloom stage in August 2019. At each sampling site, five randomly selected subsamples were equally mixed in situ to constitute a composite sample and then transported to the laboratory in a surface-sterilized ice box. Climate data at the sampling sites were acquired from the DIVA-GIS database (http://www.diva-gis.org). Location, soil type, climate factors and cropping rotation system of each sampling site are shown in Table S1.

In the laboratory, soil samples were stored at 4 °C, and a fraction of each soil composite sample was used for triplicate measurement of pH, organic matter (OM), total nitrogen (TN), available phosphorus (AP), available potassium (AK) and total salts (TS) as reported previously [21,22]. The six soil samples had 11.9–21.1 g kg^−1^ OM, 0.83–1.27 g kg^−1^ TN, 18.1–62.5 mg kg^−1^ AP, 78.0–106 mg kg^−1^ AK, 1.07 to 1.77 g kg^−1^ TS and 4.8 to 6.2 pH (Table S2). 

### 4.2. Rhizobial Isolation and Nodulation Test 

For rhizobia trapping, soil samples were mixed with sterilized vermiculite (1/5, *v/v*) in surface-sterilized plastic pots (15 cm height × 10 cm diameter) and moistened with nitrogen-free plant nutrient solution containing CaSO_4_ 0.46 g, KCl 0.075 g, MgSO_4_·7H_2_O 0.06 g, K_2_HPO_4_ 0.136 g and FeC_6_H_5_O_7_ 0.075 g in 1 L of pure water, supplemented with 1 mL of microelement solution containing ZnSO_4_ 0.22 g, MnSO_4_ 1.81 g, H_3_BO_3_ 2.86 g, CuSO_4_·5H_2_O 0.8 g and H_2_MoO_4_ 0.02 g per L of pure water. Peanut seeds of variety Yuanza 9102 purchased from Henan Academy of Agricultural Sciences were surface sterilized by immersing in 95% (*v/v*) ethanol for 30 s, followed by 2.5% (*w/v*) NaClO solution for 5 min, and then washed 8 times with sterilized water. After surface sterilization, seeds were transferred onto 0.5% water–agar in the dark at 25 °C for germination. One seedling with a root length of approximately 1 cm was sown in each pot and grown in a greenhouse for 45 days. Then, ten peanut plants were randomly uprooted from the pots of each soil sample, and four healthy mature nodules (red color inside indicating the presence of leghemoglobin) were randomly selected per plant, resulting in a total of forty nodules per soil sample (site). 

Nodules obtained by plant trapping were sterilized as described above for seeds, and rhizobia were isolated by streaking crushed nodules on yeast extract mannitol agar (YMA) [23] plates followed by incubation at 28 °C for 7−14 days. A single colony from each nodule was purified by repeated streaking on YMA plates, and all purified isolates were maintained on plates at 4 °C for temporary storage and in YM broth containing 20% (*w/v*) glycerol at −80 °C for long term storage. Nodulation ability was tested for all isolates by inoculating each of them on peanut seedlings grown in vermiculite according to the procedures of Wei et al. [24,25]. The inoculant of each isolate was prepared as a two-day culture in 5 mL YM broth at 28 °C under rotary agitation (120 rpm) until OD600 = 0.8−1.0, and the inoculation dose was 0.1 mL (10^7^−10^8^ cells) of the culture per germinated seed. Seedlings inoculated with YM broth without bacteria were included as negative controls. Seed surface sterilization, germination, cultivation, and nodule observation were as described in the trapping experiment. All isolates were authenticated as rhizobia by their ability to induce mature healthy nodules on peanut (none of the control plants nodulated) and give greater total plant dry weight than the controls.

### 4.3. PCR-Based RFLP of 16S-23S rRNA Intergenic Spacer (IGS)

Genomic DNA of each rhizobial isolate was extracted according to Terefework et al. [26] and used as the template for PCR amplification of the intergenic spacer (IGS) region with primers IGS1490 (forward) and IGS132′ (reverse) [27]. Restriction fragment length polymorphism (RFLP) of the IGS-PCR products (approximately 900 bp) was performed by digesting a 5 µL aliquot separately with each of the restriction endonucleases HaeIII, MspI or AluI. Restriction fragments were separated by electrophoresis in an agarose gel (2.5%, *w/v*) [28] containing GoldView type I nucleotide dye (Solarbio, Beijing, China, Lot No. 20140820), and results were visualized and analyzed using the DNR Bio-Imaging System (MiniBIS Pro, Jerusalem, Israel). Isolates sharing the same restriction patterns were defined as the same IGS type, and grouping analysis of IGS types was performed as previously described using the UPGMA method [29].

### 4.4. Phylogenetic Analysis of 16S rRNA Genes

Randomly selected isolates representing the different IGS types were used for 16S rRNA gene amplification with primers P1 and P6 [27] from the DNA extracts described above. The PCR products were purified and sent for commercial sequencing (Sangon Biotech Company, Shanghai, China). The acquired sequences and related sequences obtained from the GenBank database (http://www.ncbi.nlm.nih.gov/blast/Blast.cgi (accessed on 26 September 2021)) with the BLAST program were aligned using the ClustalW program in the MEGA 7.0 package [30]. To estimate the phylogenetic relationships of the strains, the phylogenetic tree was reconstructed by the maximum-likelihood (ML) method using the best-fit nucleotide substitution model defined by the Model tool test after iteration [31]. The best-fit substitution model among the 24 models implemented in MEGA 7.0 was selected based on the lowest Bayesian Information Criterion (BIC). Tree stability was estimated by bootstrapping algorithms with 1000 replicates using the MEGA 7.0 software.

### 4.5. Phylogenetic Analyses of Housekeeping and Symbiosis Genes

For representative isolates, the housekeeping genes *recA* (recombination protein A), *atpD* (ATP synthase beta chain) and *glnII* (glutamine synthetase II) were independently amplified using the primer pairs recA41F/recA640R, atpD255F/atpD782R and glnII12F/glnII689R, respectively [32,33]. In addition, sequences of type strains sharing *recA*, *atpD* or *glnII* gene similarities greater than 90% with the new isolates were extracted from the NCBI database and retained to reconstruct phylogenetic trees. The symbiotic genes *nodA* (N-acyltransferase nodulation protein A) and *nifH* (nitrogenase iron protein) were amplified for all representative isolates with primer pairs nodA-1/nodA-2 [34] and nifHF/nifHR [35], respectively, under the corresponding PCR conditions. 

All purified amplicons were commercially sequenced at Sangon Biotech Company (Shanghai, China) using the same primers as for PCR. The acquired and related sequences obtained from the GenBank database with the BLAST program, were aligned using the ClustalW program in MEGA 7.0 [30]. Model test was used to produce the best nucleotide substitution model for each alignment as described above. A maximum-likelihood phylogenetic tree with 1000 bootstrap replicates was reconstructed for each gene with the MEGA 7.0 software. The acquired sequences of *recA*, *glnII* and *atpD* from the isolates together with the corresponding gene sequences of the type strains for validated *Bradyrhizobium* species were manually concatenated and aligned using ClustalW. The concatenated gene tree was constructed with the ML method as described above.

An attempt was made to amplify the *nodC* (N-acetylglucosaminyl transferase nodulation protein C) gene of all representative isolates using the primer pairs nodCF/nodCI [35] and nodC540/nodC1160 [36], but this was unsuccessful. Instead, full genomes of selected strains were sequenced using Illumina HiSeq pair-end technology, assembled using SOAPdenovo and Falcon programs and annotated according to the NCBI Prokaryotic Genome Annotation Pipeline [37]. Complete sequences of the *nodC* genes were retrieved from the genomes of these strains deposited in the GenBank database of the National Center for Biotechnology Information (NCBI: www.ncbi.nlm.nih.gov), and they were aligned using the ClustalW program in MEGA 7.0 [30]. Model test was used to produce the best nucleotide substitution model for each alignment as described above. A maximum-likelihood phylogenetic tree with 1000 bootstrap replicates was reconstructed for each gene with the MEGA 7.0 software.

### 4.6. Genome Features

The genomes of the three novel strains were sequenced using Illumina HiSeq pair-end technology, assembled using SOAPdenovo and Falcon programs and annotated according to the NCBI Prokaryotic Genome Annotation Pipeline [37]. In this study, the whole genomes of WYCCWR 13022, WYCCWR 12699 and WYCCWR 12677 were deposited in NCBI with the accession numbers of JAUIZJ000000000, JAVTJN000000000 and JAUIZK000000000. 

## 5. Conclusions

In conclusion, diverse *Bradyrhizobium* species with similar novel symbiosis genes nodulate peanut in different provinces in China. Horizontal transfer of genes involved in nodulating peanut is common between *Bradyrhizobium* species in soils used to grow the crop in China. A novel *Bradyrhizobium* symbiovar, sv. arachis, that nodulates peanut in China is described.

## Figures and Tables

**Table 1:** Identification of genomic species by MLSA and IGS type among *Bradyrhizobium* strains isolated from *Arachis hypogaea* grown in soil from Zhengyang, Henan Province.

Representative Isolate WYCCWR no. (Field Site) ^1^	MLSA Similarity (%) with Species ^3^						IGS Type	Isolate Number (% of Total Isolates)
	*B.gd*	*B.gz*	*B.zh*	*B.gx*	*B.nn*	*B.ar*		
*Bradyrhizobium guangdongense* (Cluster 1, C1) ^2^								
13051 (ZC)	98.7	94.6	93.6	93.5	94.0	94.1	2	155 (69.82)
12658 (ZA)	100	93.1	94.4	93.7	94.1	94.2		
13038 (ZB)	98.7	94.6	93.6	93.5	94.0	94.1		
13098 (ZD)	98.7	94.6	93.6	93.5	94.0	94.1		
12802 (ZE)	100	94.9	94.4	93.7	94.1	94.2		
12718 (ZF)	98.5	94.2	93.5	93.1	93.7	93.9		
13015 (ZB)	98.7	94.6	93.6	93.5	94.0	94.1	5	5 (2.25)
12803 (ZE)	100	94.9	94.4	93.7	94.1	94.2	9	3 (1.35)
13024 (ZB)	98.5	94.2	93.5	93.1	93.7	93.9	10	2 (0.90)
12807 (ZE)	98.7	94.4	93.6	93.3	93.7	93.8	12	2 (0.90)
*Bradyrhizobium guangzhouense* (C2)								
12726 (ZF)	94.9	99.7	95.0	94.2	94.1	93.7	3	4 (1.80)
12723 (ZF)	94.9	99.7	95.0	94.2	94.1	93.7	11	2 (0.90)
*Bradyrhizobium* genosp. I (C3)								
12663 (ZA)	95.4	96.7	96.0	94.9	94.9	94.2	13	2 (0.90)
*Bradyrhizobium* genosp. II (C4)								
12677 (ZA)	94.5	94.9	94.9	94.0	94.2	94.2	4	5 (2.25)
*Bradyrhizobium zhengyangense* (C5)								
13023 (ZB)	94.4	94.9	100	94.9	94.2	94.5	1	25 (11.26)
12678 (ZA)	94.5	95.0	99.7	94.9	94.3	94.5		
12774 (ZE)	94.5	95.1	99.7	95.1	94.4	94.7		
*Bradyrhizobium* genosp. III (C6)								
13022 (ZB)	93.6	94.9	93.7	93.7	93.6	94.4	8	7 (3.15)
*Bradyrhizobium* genosp. IV (C7)								
12671 (ZA)	92.1	94.0	92.4	92.2	92.4	93.0	7	3 (1.35)
*Bradyrhizobium* genosp. V (C8)								
12669 (ZA)	92.8	94.9	94.0	94.4	93.2	94.7	6	4 (1.80)

^1^ ZA, Zhouwan, Dougou, Zhengyang county; ZB, Zhouwan, Dougou, Zhengyang county (Paddy soil); ZC, Dayu, Lanqing, Zhengyang county; ZD, Wanglaozhuang, Lanqing, Zhengyang county; ZE, Silou, Yongxing, Zhengyang county; ZF, Zhangzhai, Fuzhai, Zhengyang county; ^2^ Cluster or genomic species deduced from MLSA (concatenated *recA-atpD-glnII*) similarity values; ^3^ MLSA (concatenated *recA-glnII-atpD*) similarity values with the most closely related strains: B.gd, *Bradyrhizobium guangdongense* CCBAU 51649^T^; B.gz, *Bradyrhizobium guangzhouense* CCBAU 51670; B.zh, *Bradyrhizobium. zhengyangense* WYCCWR 13023^T^; B.gx, *Bradyrhizobium. guangxiense* CCBAU 53363^T^; B.nn, *Bradyrhizobium nanningense* CCBAU 53390^T^; B.ar, *Bradyrhizobium arachidis* CCBAU 051107^T.^

## Data Availability

All the sequences of peanut-nodulating rhizobia in this study have been submitted to GenBank and are available with the accession numbers in Figures and Supplementary Figures.

## Supplementary Materials

The following supporting information can be downloaded at: https://www.mdpi.com/article/10.3390/plants12213776/s1, Figure S1. Maximum-likelihood phylogenetic tree based on *recA* gene sequences showing the relationships of rhizobia isolated from *Arachis hypogaea* L. from Zhengyang, Hennan Province. The tree was constructed under a T92+G+I model. Bootstrap confidence values >50% are indicated at the internodes. Bar = 5% nucleotide divergence. Figure S2. Maximum-likelihood phylogenetic tree based on *glnII* gene sequences showing the relationships of rhizobia isolated from *Arachis hypogaea* L. from Zhengyang, Hennan Province. The tree was constructed under a (TN93+G+I) model. Bootstrap confidence values >50% are indicated at the internodes. Bar = 2% nucleotide divergence. Figure S3. Maximum-likelihood phylogenetic tree based on *atpD* gene sequences showing the relationships of rhizobia isolated from *Arachis hypogaea* L. from Zhengyang, Hennan Province. The tree was constructed under a T92+G+I model. Bootstrap confidence values >50% are indicated at the internodes. Bar = 5% nucleotide divergence. Figure S4. Maximum-likelihood phylogenetic tree based on *nodC* gene full sequences showing the relationships of rhizobia isolated from *Arachis hypogaea* L. from Zhengyang, Hennan Province. The tree was constructed under a T92+G model. Bootstrap confidence values >50% are indicated at the internodes. Bar = 10% nucleotide divergence. The novel sv. arachis is suggested. Table S1. Soil sample sites in Zhengyang county, Zhumadian city, Henan Province and their pedo-climatic characteristics. Table S2. Comparison of the soil physicochemical characteristics in the different sampling sites. Values are average ± standard deviation of three sub-samples.
