# Supplementary material for: Diverse Bradyrhizobium spp. with Similar Symbiosis Genes Nodulate Peanut in Different Regions of China: Characterization of Symbiovar sv. Arachis
Source: Plants (Basel). 2023 Nov 6;12(21):3776. doi: 10.3390/plants12213776 (PMC10647606; doi:10.3390/plants12213776)
Supplement: Supplementary file 1 [file plants-12-03776-s001.zip › Suppl. Figures 1003.pptx]

## Slide 1
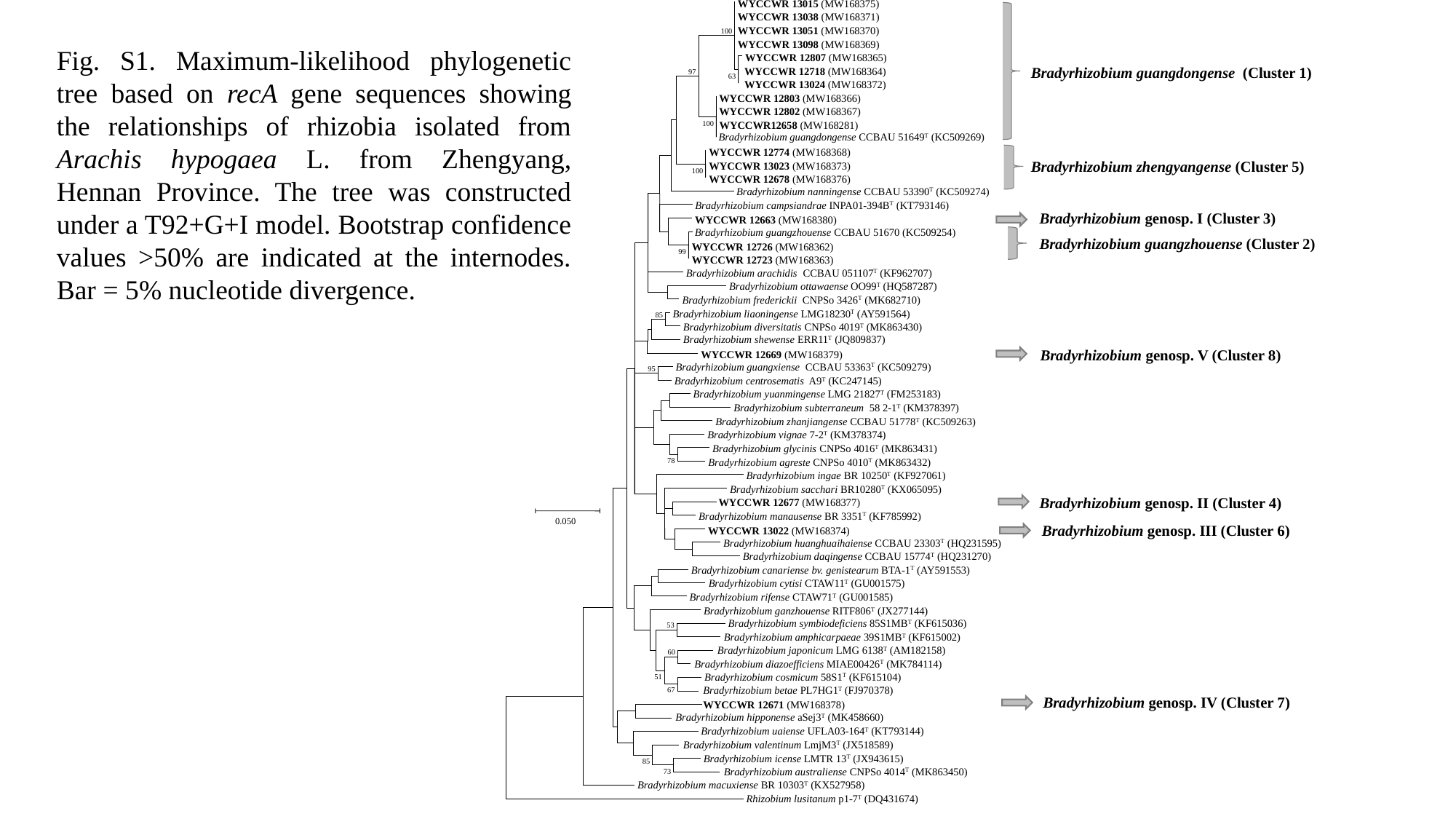

WYCCWR 13015 (MW168375)
 WYCCWR 13038 (MW168371)
 WYCCWR 13051 (MW168370)
100
 WYCCWR 13098 (MW168369)
 WYCCWR 12807 (MW168365)
 WYCCWR 12718 (MW168364)
97
63
 WYCCWR 13024 (MW168372)
 WYCCWR 12803 (MW168366)
 WYCCWR 12802 (MW168367)
100
 WYCCWR12658 (MW168281)
 Bradyrhizobium guangdongense CCBAU 51649T (KC509269)
 WYCCWR 12774 (MW168368)
 WYCCWR 13023 (MW168373)
100
 WYCCWR 12678 (MW168376)
 Bradyrhizobium nanningense CCBAU 53390T (KC509274)
 Bradyrhizobium campsiandrae INPA01-394BT (KT793146)
 WYCCWR 12663 (MW168380)
 Bradyrhizobium guangzhouense CCBAU 51670 (KC509254)
 WYCCWR 12726 (MW168362)
99
 WYCCWR 12723 (MW168363)
 Bradyrhizobium arachidis CCBAU 051107T (KF962707)
 Bradyrhizobium ottawaense OO99T (HQ587287)
 Bradyrhizobium frederickii CNPSo 3426T (MK682710)
 Bradyrhizobium liaoningense LMG18230T (AY591564)
85
 Bradyrhizobium diversitatis CNPSo 4019T (MK863430)
 Bradyrhizobium shewense ERR11T (JQ809837)
 WYCCWR 12669 (MW168379)
 Bradyrhizobium guangxiense CCBAU 53363T (KC509279)
95
 Bradyrhizobium centrosematis A9T (KC247145)
 Bradyrhizobium yuanmingense LMG 21827T (FM253183)
 Bradyrhizobium subterraneum 58 2-1T (KM378397)
 Bradyrhizobium zhanjiangense CCBAU 51778T (KC509263)
 Bradyrhizobium vignae 7-2T (KM378374)
 Bradyrhizobium glycinis CNPSo 4016T (MK863431)
 Bradyrhizobium agreste CNPSo 4010T (MK863432)
78
 Bradyrhizobium ingae BR 10250T (KF927061)
 Bradyrhizobium sacchari BR10280T (KX065095)
 WYCCWR 12677 (MW168377)
 Bradyrhizobium manausense BR 3351T (KF785992)
 WYCCWR 13022 (MW168374)
 Bradyrhizobium huanghuaihaiense CCBAU 23303T (HQ231595)
 Bradyrhizobium daqingense CCBAU 15774T (HQ231270)
 Bradyrhizobium canariense bv. genistearum BTA-1T (AY591553)
 Bradyrhizobium cytisi CTAW11T (GU001575)
 Bradyrhizobium rifense CTAW71T (GU001585)
 Bradyrhizobium ganzhouense RITF806T (JX277144)
 Bradyrhizobium symbiodeficiens 85S1MBT (KF615036)
53
 Bradyrhizobium amphicarpaeae 39S1MBT (KF615002)
 Bradyrhizobium japonicum LMG 6138T (AM182158)
60
 Bradyrhizobium diazoefficiens MIAE00426T (MK784114)
 Bradyrhizobium cosmicum 58S1T (KF615104)
51
 Bradyrhizobium betae PL7HG1T (FJ970378)
67
 WYCCWR 12671 (MW168378)
 Bradyrhizobium hipponense aSej3T (MK458660)
 Bradyrhizobium uaiense UFLA03-164T (KT793144)
 Bradyrhizobium valentinum LmjM3T (JX518589)
 Bradyrhizobium icense LMTR 13T (JX943615)
85
 Bradyrhizobium australiense CNPSo 4014T (MK863450)
73
 Bradyrhizobium macuxiense BR 10303T (KX527958)
 Rhizobium lusitanum p1-7T (DQ431674)
0.050
Fig. S1. Maximum-likelihood phylogenetic tree based on recA gene sequences showing the relationships of rhizobia isolated from Arachis hypogaea L. from Zhengyang, Hennan Province. The tree was constructed under a T92+G+I model. Bootstrap confidence values >50% are indicated at the internodes. Bar = 5% nucleotide divergence.
Bradyrhizobium guangdongense (Cluster 1)
Bradyrhizobium zhengyangense (Cluster 5)
Bradyrhizobium genosp. I (Cluster 3)
Bradyrhizobium guangzhouense (Cluster 2)
Bradyrhizobium genosp. V (Cluster 8)
Bradyrhizobium genosp. II (Cluster 4)
Bradyrhizobium genosp. III (Cluster 6)
Bradyrhizobium genosp. IV (Cluster 7)

## Slide 2
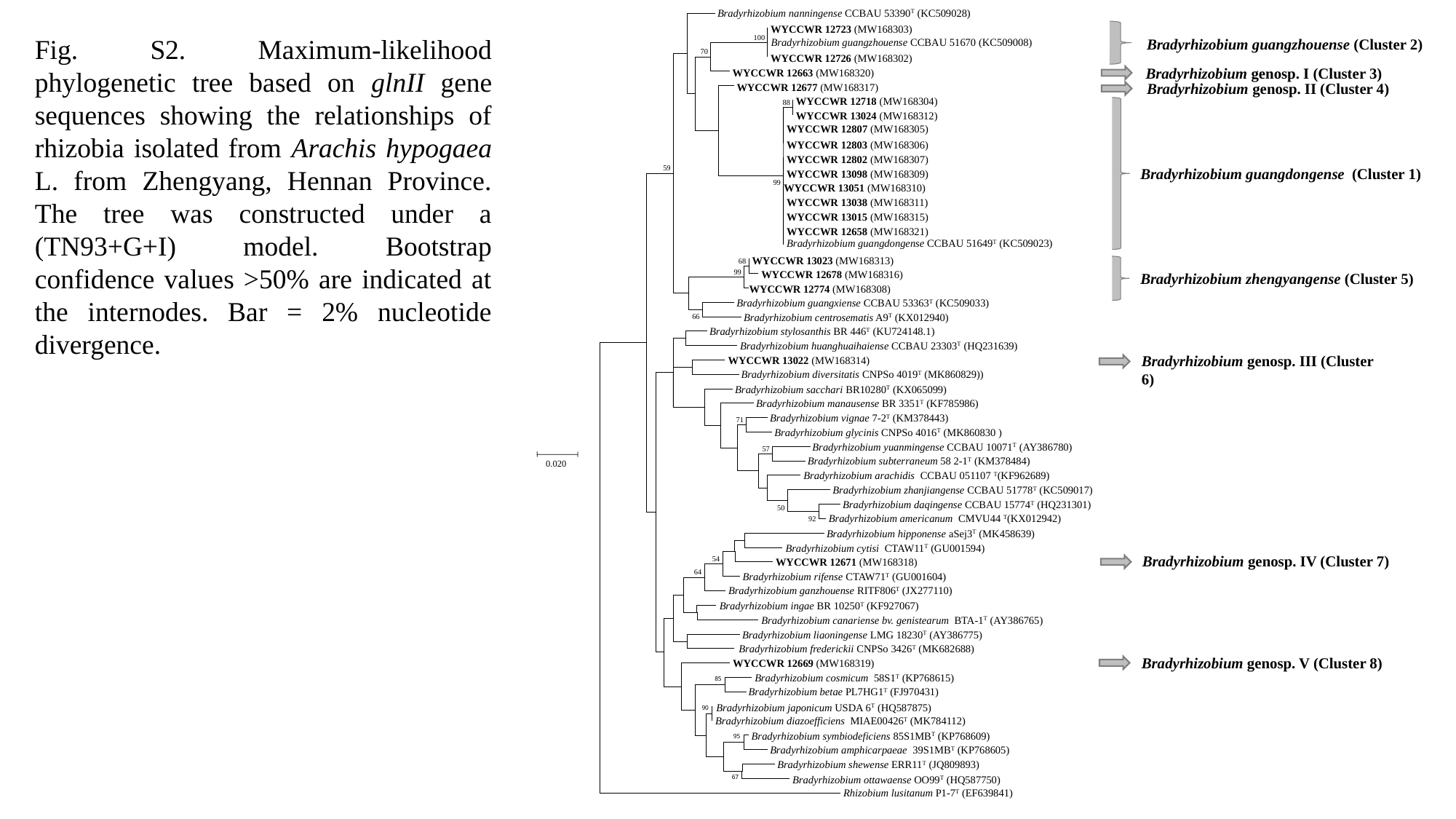

Bradyrhizobium nanningense CCBAU 53390T (KC509028)
 WYCCWR 12723 (MW168303)
100
Bradyrhizobium guangzhouense CCBAU 51670 (KC509008)
70
 WYCCWR 12726 (MW168302)
 WYCCWR 12663 (MW168320)
 WYCCWR 12677 (MW168317)
 WYCCWR 12718 (MW168304)
88
 WYCCWR 13024 (MW168312)
 WYCCWR 12807 (MW168305)
 WYCCWR 12803 (MW168306)
 WYCCWR 12802 (MW168307)
59
 WYCCWR 13098 (MW168309)
99
WYCCWR 13051 (MW168310)
 WYCCWR 13038 (MW168311)
 WYCCWR 13015 (MW168315)
 WYCCWR 12658 (MW168321)
 Bradyrhizobium guangdongense CCBAU 51649T (KC509023)
 WYCCWR 13023 (MW168313)
68
99
 WYCCWR 12678 (MW168316)
WYCCWR 12774 (MW168308)
 Bradyrhizobium guangxiense CCBAU 53363T (KC509033)
 Bradyrhizobium centrosematis A9T (KX012940)
66
 Bradyrhizobium stylosanthis BR 446T (KU724148.1)
 Bradyrhizobium huanghuaihaiense CCBAU 23303T (HQ231639)
 WYCCWR 13022 (MW168314)
 Bradyrhizobium diversitatis CNPSo 4019T (MK860829))
 Bradyrhizobium sacchari BR10280T (KX065099)
 Bradyrhizobium manausense BR 3351T (KF785986)
 Bradyrhizobium vignae 7-2T (KM378443)
71
 Bradyrhizobium glycinis CNPSo 4016T (MK860830 )
 Bradyrhizobium yuanmingense CCBAU 10071T (AY386780)
57
 Bradyrhizobium subterraneum 58 2-1T (KM378484)
 Bradyrhizobium arachidis CCBAU 051107 T(KF962689)
 Bradyrhizobium zhanjiangense CCBAU 51778T (KC509017)
 Bradyrhizobium daqingense CCBAU 15774T (HQ231301)
50
 Bradyrhizobium americanum CMVU44 T(KX012942)
92
 Bradyrhizobium hipponense aSej3T (MK458639)
 Bradyrhizobium cytisi CTAW11T (GU001594)
54
 WYCCWR 12671 (MW168318)
64
 Bradyrhizobium rifense CTAW71T (GU001604)
Bradyrhizobium ganzhouense RITF806T (JX277110)
 Bradyrhizobium ingae BR 10250T (KF927067)
 Bradyrhizobium canariense bv. genistearum BTA-1T (AY386765)
 Bradyrhizobium liaoningense LMG 18230T (AY386775)
 Bradyrhizobium frederickii CNPSo 3426T (MK682688)
 WYCCWR 12669 (MW168319)
 Bradyrhizobium cosmicum 58S1T (KP768615)
85
 Bradyrhizobium betae PL7HG1T (FJ970431)
 Bradyrhizobium japonicum USDA 6T (HQ587875)
90
 Bradyrhizobium diazoefficiens MIAE00426T (MK784112)
 Bradyrhizobium symbiodeficiens 85S1MBT (KP768609)
95
 Bradyrhizobium amphicarpaeae 39S1MBT (KP768605)
 Bradyrhizobium shewense ERR11T (JQ809893)
 Bradyrhizobium ottawaense OO99T (HQ587750)
67
 Rhizobium lusitanum P1-7T (EF639841)
0.020
Fig. S2. Maximum-likelihood phylogenetic tree based on glnII gene sequences showing the relationships of rhizobia isolated from Arachis hypogaea L. from Zhengyang, Hennan Province. The tree was constructed under a (TN93+G+I) model. Bootstrap confidence values >50% are indicated at the internodes. Bar = 2% nucleotide divergence.
Bradyrhizobium guangzhouense (Cluster 2)
Bradyrhizobium genosp. I (Cluster 3)
Bradyrhizobium genosp. II (Cluster 4)
Bradyrhizobium guangdongense (Cluster 1)
Bradyrhizobium zhengyangense (Cluster 5)
Bradyrhizobium genosp. III (Cluster 6)
Bradyrhizobium genosp. IV (Cluster 7)
Bradyrhizobium genosp. V (Cluster 8)

## Slide 3
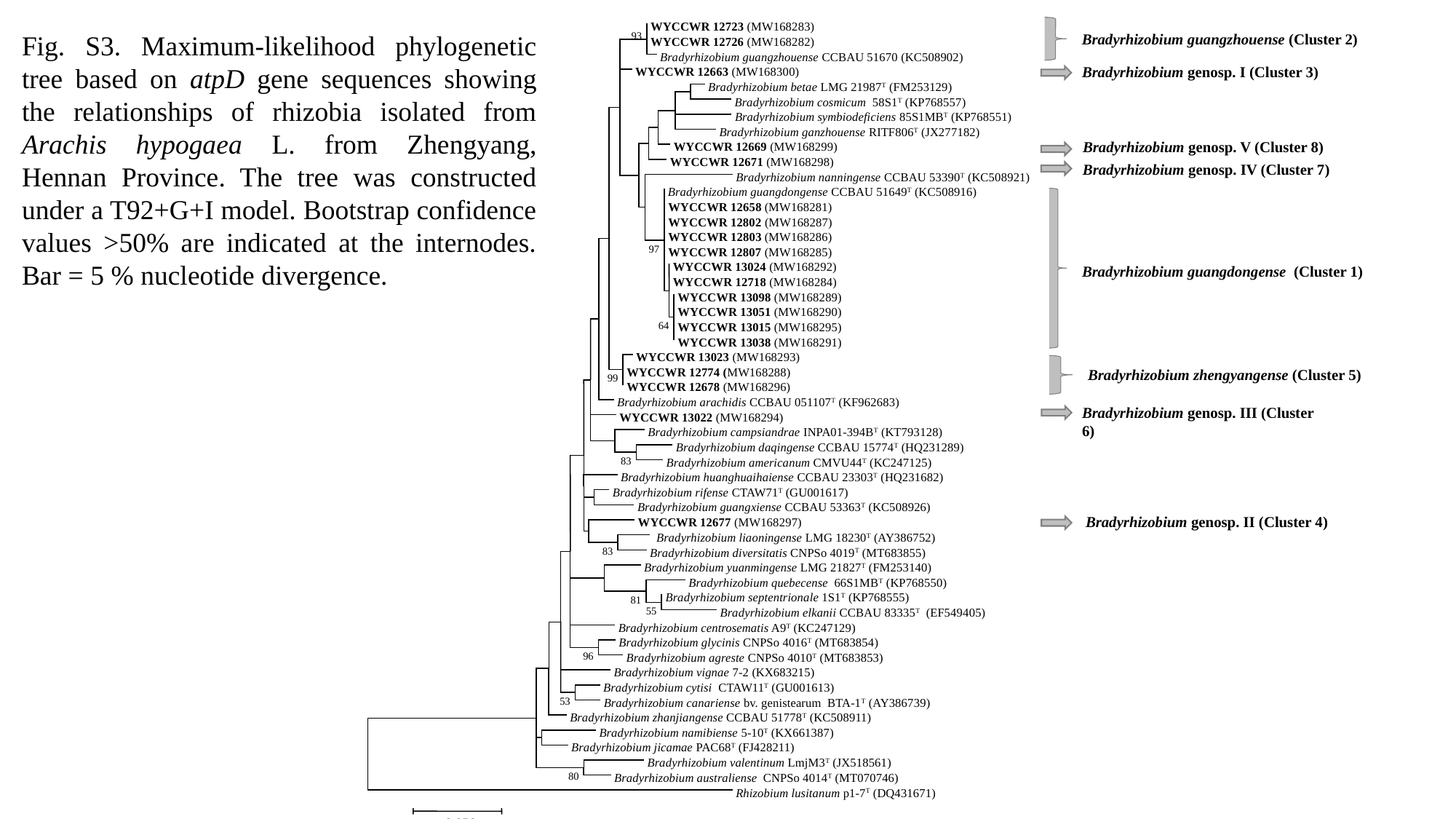

WYCCWR 12723 (MW168283)
93
 WYCCWR 12726 (MW168282)
 Bradyrhizobium guangzhouense CCBAU 51670 (KC508902)
 WYCCWR 12663 (MW168300)
 Bradyrhizobium betae LMG 21987T (FM253129)
 Bradyrhizobium cosmicum 58S1T (KP768557)
 Bradyrhizobium symbiodeficiens 85S1MBT (KP768551)
 Bradyrhizobium ganzhouense RITF806T (JX277182)
 WYCCWR 12669 (MW168299)
 WYCCWR 12671 (MW168298)
 Bradyrhizobium nanningense CCBAU 53390T (KC508921)
 Bradyrhizobium guangdongense CCBAU 51649T (KC508916)
 WYCCWR 12658 (MW168281)
 WYCCWR 12802 (MW168287)
 WYCCWR 12803 (MW168286)
97
 WYCCWR 12807 (MW168285)
 WYCCWR 13024 (MW168292)
 WYCCWR 12718 (MW168284)
 WYCCWR 13098 (MW168289)
 WYCCWR 13051 (MW168290)
64
 WYCCWR 13015 (MW168295)
 WYCCWR 13038 (MW168291)
 WYCCWR 13023 (MW168293)
 WYCCWR 12774 (MW168288)
99
 WYCCWR 12678 (MW168296)
 Bradyrhizobium arachidis CCBAU 051107T (KF962683)
 WYCCWR 13022 (MW168294)
 Bradyrhizobium campsiandrae INPA01-394BT (KT793128)
 Bradyrhizobium daqingense CCBAU 15774T (HQ231289)
83
 Bradyrhizobium americanum CMVU44T (KC247125)
 Bradyrhizobium huanghuaihaiense CCBAU 23303T (HQ231682)
 Bradyrhizobium rifense CTAW71T (GU001617)
 Bradyrhizobium guangxiense CCBAU 53363T (KC508926)
 WYCCWR 12677 (MW168297)
 Bradyrhizobium liaoningense LMG 18230T (AY386752)
83
 Bradyrhizobium diversitatis CNPSo 4019T (MT683855)
 Bradyrhizobium yuanmingense LMG 21827T (FM253140)
 Bradyrhizobium quebecense 66S1MBT (KP768550)
 Bradyrhizobium septentrionale 1S1T (KP768555)
81
55
 Bradyrhizobium elkanii CCBAU 83335T (EF549405)
 Bradyrhizobium centrosematis A9T (KC247129)
 Bradyrhizobium glycinis CNPSo 4016T (MT683854)
96
 Bradyrhizobium agreste CNPSo 4010T (MT683853)
 Bradyrhizobium vignae 7-2 (KX683215)
 Bradyrhizobium cytisi CTAW11T (GU001613)
53
 Bradyrhizobium canariense bv. genistearum BTA-1T (AY386739)
 Bradyrhizobium zhanjiangense CCBAU 51778T (KC508911)
 Bradyrhizobium namibiense 5-10T (KX661387)
 Bradyrhizobium jicamae PAC68T (FJ428211)
 Bradyrhizobium valentinum LmjM3T (JX518561)
80
 Bradyrhizobium australiense CNPSo 4014T (MT070746)
 Rhizobium lusitanum p1-7T (DQ431671)
0.050
Fig. S3. Maximum-likelihood phylogenetic tree based on atpD gene sequences showing the relationships of rhizobia isolated from Arachis hypogaea L. from Zhengyang, Hennan Province. The tree was constructed under a T92+G+I model. Bootstrap confidence values >50% are indicated at the internodes. Bar = 5 % nucleotide divergence.
Bradyrhizobium guangzhouense (Cluster 2)
Bradyrhizobium genosp. I (Cluster 3)
Bradyrhizobium genosp. V (Cluster 8)
Bradyrhizobium genosp. IV (Cluster 7)
Bradyrhizobium guangdongense (Cluster 1)
Bradyrhizobium zhengyangense (Cluster 5)
Bradyrhizobium genosp. III (Cluster 6)
Bradyrhizobium genosp. II (Cluster 4)

## Slide 4
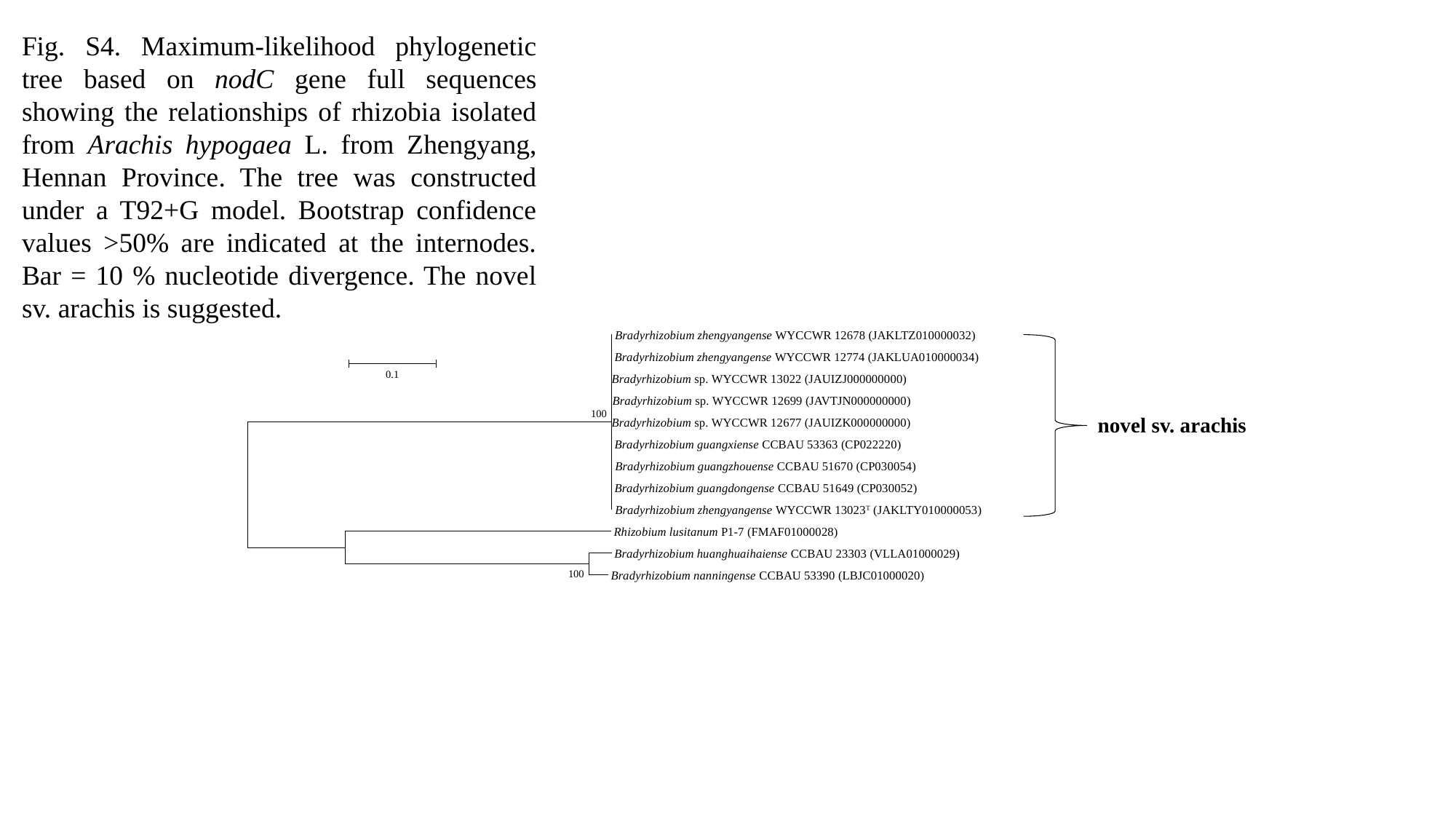

Fig. S4. Maximum-likelihood phylogenetic tree based on nodC gene full sequences showing the relationships of rhizobia isolated from Arachis hypogaea L. from Zhengyang, Hennan Province. The tree was constructed under a T92+G model. Bootstrap confidence values >50% are indicated at the internodes. Bar = 10 % nucleotide divergence. The novel sv. arachis is suggested.
 Bradyrhizobium zhengyangense WYCCWR 12678 (JAKLTZ010000032)
 Bradyrhizobium zhengyangense WYCCWR 12774 (JAKLUA010000034)
0.1
Bradyrhizobium sp. WYCCWR 13022 (JAUIZJ000000000)
Bradyrhizobium sp. WYCCWR 12699 (JAVTJN000000000)
100
Bradyrhizobium sp. WYCCWR 12677 (JAUIZK000000000)
 Bradyrhizobium guangxiense CCBAU 53363 (CP022220)
Bradyrhizobium guangzhouense CCBAU 51670 (CP030054)
 Bradyrhizobium guangdongense CCBAU 51649 (CP030052)
 Bradyrhizobium zhengyangense WYCCWR 13023T (JAKLTY010000053)
 Rhizobium lusitanum P1-7 (FMAF01000028)
 Bradyrhizobium huanghuaihaiense CCBAU 23303 (VLLA01000029)
100
 Bradyrhizobium nanningense CCBAU 53390 (LBJC01000020)
novel sv. arachis
