# Supplementary material for: Diverse Bradyrhizobium spp. with Similar Symbiosis Genes Nodulate Peanut in Different Regions of China: Characterization of Symbiovar sv. Arachis
Source: Plants (Basel). 2023 Nov 6;12(21):3776. doi: 10.3390/plants12213776 (PMC10647606; doi:10.3390/plants12213776)
Supplement: Supplementary file 1 [file plants-12-03776-s001.zip › SupplementaryTables 1003.pdf]

Table S1. Soil sample sites in Zhengyang county, Zhumadian city, Henan Province and their pedo-climatic characteristics*

Code site	Sampling sites	Soil type	Longitude and Latitude	Altitude (m)	Sampling date	Average T_{min} °C	Average T_{max} °C	Precipitation (mm)	Crop rotation for sampling sites
ZA	Zhouwan village, Dougou town	Fluvo-aquic soil	N32°22′23.8512″ E114°18′59.8536″	51.9	2019.5.28	21.4	30.1	145.0	wheat-peanut
ZB	Zhouwan village, Dougou town	Paddy soil	N32°22′41.286″ E114°19′3.6876″	50.3	2019.5.28	21.4	30.1	145.0	wheat-peanut/ rice-green manure
ZC	Dayu village, Lanqing town	Shajiang black soil	N32°27′37.1736″ E114°20′11.5656″	67.8	2019.5.29	21.3	30.2	128.5	wheat-peanut
ZD	Wanglaozhuang village, Lanqing town	Yellow cinnamon soil	N32°27′33.0192″ E114°20′6.0432″	74.9	2019.5.24	21.3	30.2	128.5	wheat-peanut
ZE	Silou village, Yongxing town	Yellow cinnamon soil	N32°28′57.0504″ E114°29′30.5196″	60.2	2019.5.28	21.3	30.3	128.0	wheat-peanut
ZF	Zhangzhai village, Fuzhai town	Yellow cinnamon soil	N32°40′52.23″ E114°22′56.2224″	73.3	2019.5.24	21.1	30.2	116.8	wheat-peanut

*. Average T_{min}: average of daily minimum temperatures from June to September 2019 for each site. Average T_{max}: average of daily maximum temperatures from June to September 2019 for each site. Precipitation: total precipitations from June to September 2019 for each site.

Table S2. Comparison of the soil physicochemical characteristics in the different sampling sites. Values are average ± standard deviation of three sub-samples.

Sampling sites #	Soil traits					
	Organic matter g kg^{-1}	Total N g kg^{-1}	Available P mg kg^{-1}	Available K mg kg^{-1}	pH (water)	Total salts g kg^{-1}
ZA	11.93±1.11	0.83±0.01	62.50±1.31	78.00±0.00	4.90±0.10	1.30±0.20
ZB	20.40±0.53	1.20±0.03	18.07±0.45	97.33±2.31	6.20±0.00	1.27±0.23
ZC	16.47±0.48	1.00±0.04	23.47±2.02	96.00±0.00	4.90±0.10	1.23±0.058
ZD	18.47±1.10	1.09±0.04	23.90±0.35	92.00±0.00	4.80±0.00	1.50±0.10
ZE	21.13±0.35	1.27±0.02	35.27±3.01	106.33±4.04	4.80±0.00	1.07±0.30
ZF	15.70±0.17	0.95±0.05	34.47±2.33	98.67±2.31	5.47±0.15	1.77±0.40

One-year rotation (wheat-peanut) except site ZB with a two-year rotation (wheat-peanut and rice-green manure). Code of site: ZA, Zhouwan, Dougou, Zhengyang county；ZB，Zhouwan, Dougou, Zhengyang county；ZC，Dayu, Lanqing, Zhengyang county；ZD，Wanglaozhuang, Lanqing, Zhengyang county；ZE，Silou, Yongxing, Zhengyang county；ZF，Zhangzhai, Fuzhai, Zhengyang county
